# Extracellular vesicle transfer of miR-1 to adipose tissue modifies lipolytic pathways following resistance exercise

**DOI:** 10.1172/jci.insight.182589

**Published:** 2024-11-08

**Authors:** Benjamin I. Burke, Ahmed Ismaeel, Douglas E. Long, Lauren A. Depa, Peyton T. Coburn, Jensen Goh, Tolulope P. Saliu, Bonnie J. Walton, Ivan J. Vechetti, Bailey D. Peck, Taylor R. Valentino, C. Brooks Mobley, Hasiyet Memetimin, Dandan Wang, Brian S. Finlin, Philip A. Kern, Charlotte A. Peterson, John J. McCarthy, Yuan Wen

**Affiliations:** 1 Department of Physiology, College of Medicine,; 2 Center for Muscle Biology, College of Health Sciences,; 3 Division of Endocrinology, Department of Internal Medicine, College of Medicine,; 4 Department of Biostatistics, College of Public Health, and; 5 Division of Biomedical Informatics, Department of Internal Medicine, College of Medicine, University of Kentucky, Lexington, Kentucky, USA.

**Keywords:** Metabolism, Muscle biology, Adipose tissue, Skeletal muscle, Transport

## Abstract

Extracellular vesicles (EVs) have emerged as important mediators of intertissue signaling and exercise adaptations. In this human study, we provide evidence that muscle-specific microRNA-1 (miR-1) was transferred to adipose tissue via EVs following an acute bout of resistance exercise. Using a multimodel machine learning automation tool, we discovered muscle primary miR-1 transcript and CD63^+^ EV count in circulation as top explanatory features for changes in adipose miR-1 levels in response to resistance exercise. RNA-Seq and in-silico prediction of miR-1 target genes identified caveolin 2 (*CAV2*) and tripartite motif containing 6 (*TRIM6*) as miR-1 target genes downregulated in the adipose tissue of a subset of participants with the highest increases in miR-1 levels following resistance exercise. Overexpression of miR-1 in differentiated human adipocyte-derived stem cells downregulated these miR-1 targets and enhanced catecholamine-induced lipolysis. These data identify a potential EV-mediated mechanism by which skeletal muscle communicates with adipose tissue and modulates lipolysis via miR-1.

## Abstract 2

Upon exercise, a muscle-specific microRNA is packaged into vesicles and delivered to adipose tissue, where it enhances lipid breakdown.

## Graphical abstract

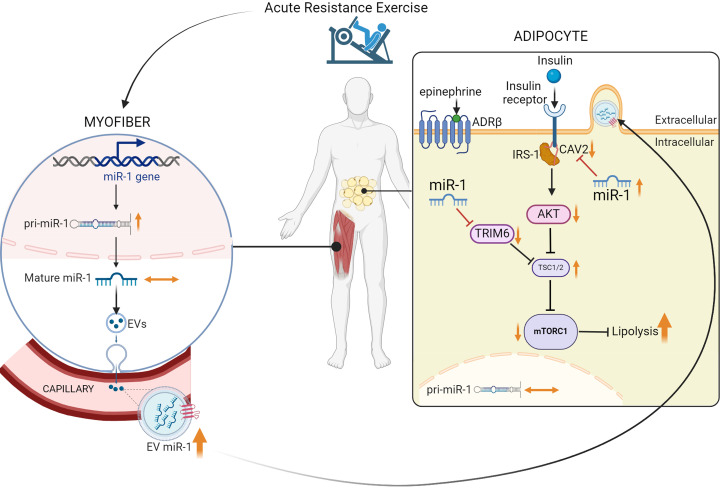

## Introduction

Extracellular vesicles (EVs) are cell-derived endosomal or plasma membrane-bound structures released from cells (1). Although initially thought to be a mechanism to rid cells of unneeded or unwanted material, it has since been found that EVs are also an important means of intercellular communication, facilitating the transfer of various bioactive molecules, including microRNAs (miRs), between cells and tissues (2–7). In addition to being the largest organ in the human body, skeletal muscle also acts as an endocrine tissue, which may regulate the metabolism of other tissues through secretion of myokines, as well as other cargo carried by EVs (8). Because of the heightened skeletal muscle metabolism and activity during exercise, a number of studies have assessed the dynamics of EV release following various exercise interventions. These studies show that circulating EVs are increased following an acute bout of exercise in both animal models and in human participants (9–16). Studies have also demonstrated that intertissue signaling via EVs may contribute to exercise adaptations (17, 18). Using a mouse model of resistance exercise, we previously reported that skeletal muscle communicates with adipose tissue through EV-delivery of miR-1 (18), a conserved, highly expressed muscle-specific miR (19). Upon delivery, miR-1 promoted adrenergic signaling and lipolysis in adipose tissue. We also found that in human research participants, miR-1 levels were reduced in skeletal muscle 30 minutes after an acute bout of resistance exercise, accompanied by an increased abundance of miR-1 in plasma EVs (18).

In this study, we found that muscle-specific miR-1 increases in circulating EVs and adipose tissue in response to acute resistance exercise concurrent with increased miR-1 production in skeletal muscle, suggesting a pathway of inter-organ communication mediated by EVs. In adipose tissue, miR-1 acts upon several target genes to modulate lipid metabolism through enhanced catecholamine-induced lipolysis. Overall, these findings provide evidence of EV-mediated cross-tissue communication between skeletal muscle and adipose tissue in humans, suggesting a potential mechanism by which exercise may influence adipose tissue metabolism.

## Results

### Acute resistance exercise in humans induces increased production of skeletal muscle miR-1 concurrent with increased miR-1 in EVs and adipose tissue.

**Figure 1:**
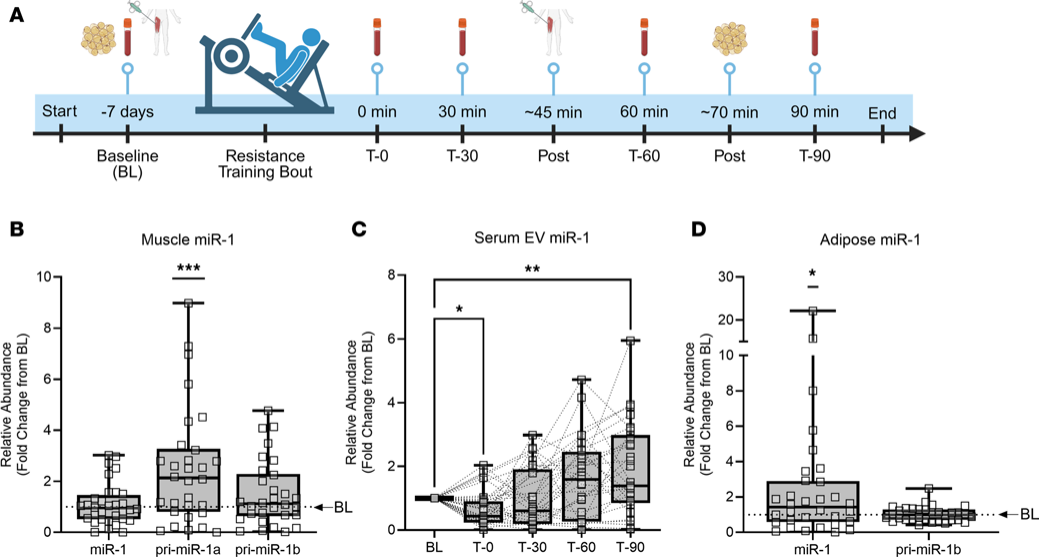
Acute resistance exercise in humans induces increased production of skeletal muscle miR-1 concurrent with increased miR-1 in EVs and adipose tissue. (**A**) Schematic diagram showing timeline of skeletal muscle and adipose tissue biopsies and blood draws relative to the acute resistance exercise bout. Expression of mature miR-1 and primary transcripts of miR-1 (pri-miR-1a, pri-miR-1b) in (**B**) skeletal muscle (*n* = 32), (**C**) serum EVs (mature miR-1 only; *n* = 31), and (**D**) adipose tissue relative to baseline levels (BL; denoted by the dotted line; *n* = 32). Data are expressed with min-to-max box plots and were compared using 1-sample Wilcoxon *t* tests (skeletal muscle and adipose tissue) or a 1-way ANOVA with Dunnett corrections for multiple comparisons (serum EVs). Serum EV miR-1 outliers were removed using the ROUT method (Q = 5%). *P < 0.05; **P < 0.01; ***P < 0.001.

Previously, we found that in response to mechanical overload, a model of resistance exercise in mice, EV-delivered skeletal muscle miR-1 enhanced catecholamine sensitivity in adipose tissue (18). Additionally, in a small sample of human participants, an acute bout of lower body resistance training (i.e., leg press and knee extension) led to reduced skeletal muscle miR-1, with a concomitant increase of miR-1 levels in plasma EVs 30 minutes after exercise (18). Thus, we sought to further explore the potential axis of EV-mediated communication between skeletal muscle and adipose tissue in humans, with both skeletal muscle and adipose tissue biopsies and blood draws at several time points. To this end, we performed the study outlined in Figure 1A, where blood draws, muscle, and adipose tissue biopsies were taken at indicated time points. Participant characteristics are described in Supplemental Table 1; supplemental material available online with this article; https://doi.org/10.1172/jci.insight.182589DS1 Baseline blood draw and biopsies (BL) were carried out approximately 1 week prior to a high-intensity, whole-body resistance exercise bout. Blood was drawn immediately (T-0), 30 (T-30), 60 (T-60), and 90 (T-90) minutes after exercise. Biopsies of skeletal muscle and subcutaneous adipose tissue were taken approximately 45 and 70 minutes after exercise (post), respectively. The expression of miR-1 did not change in skeletal muscle after exercise relative to BL; however, the expression of miR-1 primary transcript A (pri-miR-1a), one of the primary transcripts for miR-1, was significantly increased 2.5-fold (*P* < 0.001) (Figure 1B). Increased production of primary miR-1 transcript without a change in the levels of the mature form of miR-1 suggests increased export of miR-1 from skeletal muscle after exercise. To further confirm release of miR-1 into circulation, we measured levels of serum EV-associated miR-1 using exoEasy membrane-affinity purification at all 4 time points after the acute resistance bout, normalized to BL. After an initial significant decrease at T-0 (*P* < 0.01), circulating EV miR-1 levels were significantly increased by T-90 (*P* < 0.01) (Figure 1C). In addition, there was a concomitant significant increase in adipose miR-1 levels (*P*= 0.02) after exercise relative to BL, without any change in pri-miR-1b and no detection of pri-miR-1a (Figure 1D). Taken together, these results support a mechanism of miR-1 export from skeletal muscle into circulation by EVs in response to an acute bout of resistance exercise in humans. The data also demonstrate an increase in mature muscle-specific miR-1 levels in adipose tissue, potentially via EV-mediated uptake.

### Acute resistance exercise produces few alterations in EV size and population composition in humans.

To characterize the EV phenotype in response to an acute bout of resistance exercise in humans, we utilized single-particle interferometric reflectance imaging sensor coupled with immunocapture and immunofluorescence (ExoView) technology to comprehensively characterize the changes in serum EV profiles at BL and T-30. The 30-minute time point was chosen because several myokines and muscle-derived factors have been previously shown to peak in circulation approximately 30 minutes after acute exercise training (20–22). The workflow for the protein-capture ExoView platform is outlined in Supplemental Figure 1A; using specific fluorochrome-conjugated antibodies targeting the canonical tetraspanin EV markers, CD63, CD81, and CD9 allowed for direct analysis at a single EV level of detection (23). The ExoView analyses revealed few alterations in overall percentage of tetraspanin-labeled EVs or EV particle size at T-30 (Supplemental Figure 1, B–D), possibly due to high variability among participants. A small but significant increase in the proportion of EVs labeled with only CD9 (*P* = 0.04) (Supplemental Figure 1B) and an increase in the particle size of EVs labeled with only CD81 (*P* = 0.02) (Supplemental Figure 1C) were observed at T-30 relative to BL.

### Sex and adiposity alter EV response to acute resistance exercise.

Studies have suggested that adiposity and sex may influence EV characteristics including particle size and tetraspanin profiles (24). Thus, we sought to investigate interactions of the factors of sex and adiposity (body fat percentage; BF%) with the EV characteristics following an acute bout of resistance exercise. We separated individuals with high and low BF% according to the following criteria: males: > 30% (*n* = 7) and < 30% (*n* = 10), respectively; females: > 40% (*n* = 7) and < 40% (*n* = 5), respectively. There were significant interaction effects of exercise and sex on the number of total (*P* = 0.05, Supplemental Figure 2A) and CD9/CD63 double-positive particles (*P* = 0.04, Supplemental Figure 2B), along with trending interaction effects of exercise and sex on CD9-only (*P* = 0.06, Supplemental Figure 2C) and CD9/CD63/CD81 triple-labeled particle number (*P* = 0.09, Supplemental Figure 2D). There was also a significant interaction effect of exercise and BF% on the number of CD9/CD63 double-positive particles (*P* = 0.02, Supplemental Figure 2B) and a trending interaction effect of exercise and BF% on the number of C63-only particles (*P* = 0.07, Supplemental Figure 2E). There were no interaction effects of exercise and sex or BF% on particle counts for CD81-only, CD9/CD81 double-labeled, or CD63/CD81 double-labeled particles (Supplemental Figure 2, F–H). These data demonstrate for the first time that EV particle size and the serum composition of EV subtypes are impacted by resistance exercise and may be influenced by sex and adiposity. Notably, it appears that many of the interaction effects described above are driven by the male high-BF% group.

### Machine learning identifies CD63 EVs as a major contributor to exercise-induced changes in adipose miR-1.

**Figure 2:**
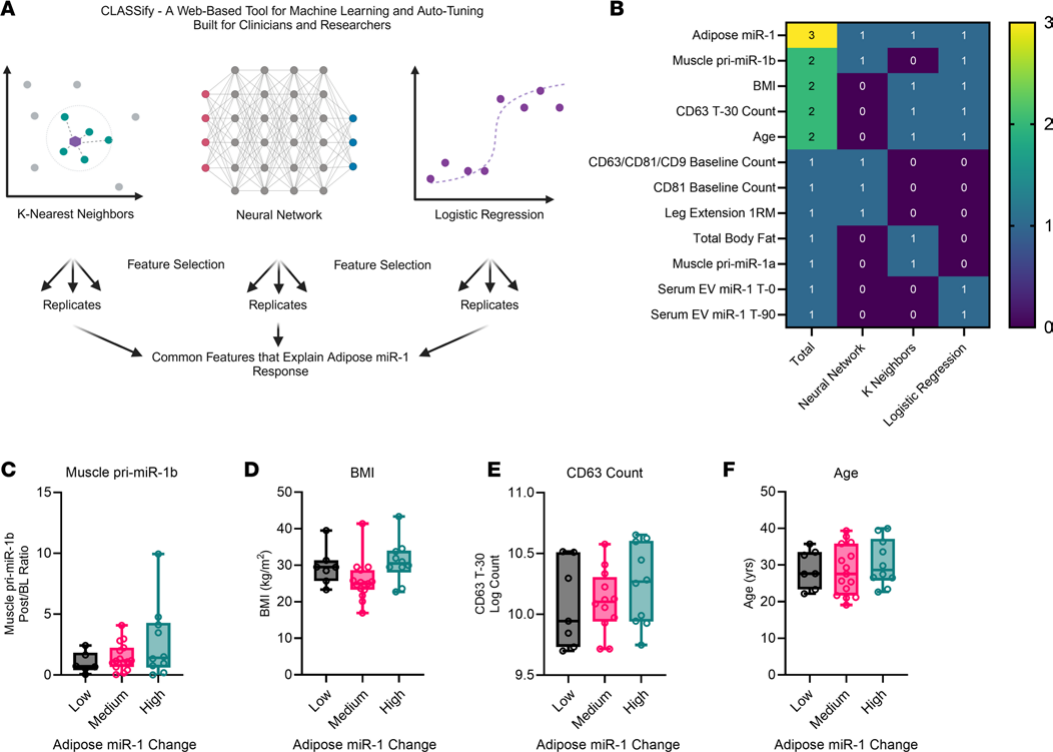
Machine learning identifies CD63 EVs as a major contributor to exercise-induced changes in adipose miR-1. (**A**) Schematic diagram outlining the workflow of CLASSify. (**B**) Nonlinear predictors of the exercise-induced changes in miR-1 levels in adipose tissue identified by 1 or more of the 3 nonparametric models: K-Nearest Neighbors, Neural Network, and Logistic Regression. Participants were divided into low (< 0.5-fold change; *n* = 7), medium (0.5–2.0-fold change; *n* = 15), and high (> 2.0-fold change; *n* = 10) miR-1 based on changes in adipose miR-1 in response to acute exercise. The relationship among exercise-induced changes in adipose miR-1 and (**C**) muscle miR-1 primary transcript 1b (pri-miR-1b), (**D**) BMI, (**E**) CD63 count, and (**F**) age. Data are expressed with min-to-max box plots. BMI, body mass index; BL, baseline; T-0, 0-minute time point; T-30, 30-minute time point; T-90, 90-minute time point.

We next interrogated the relationship between changes in miR-1 levels in serum EVs, adipose, and muscle tissue with potential predictors such as age, sex, BMI, visceral fat mass, and measures of muscle mass (Supplemental Table 2). Forward step-wise linear regression analyses did not identify any significant statistical predictors of miR-1 changes in serum EVs, adipose, or muscle. Paired with our observed variability in the response among participants for most of our measurements (e.g., miR-1 changes and EVs tetraspanin abundance), these findings suggested to us several possibilities, including limitations of statistical assumptions, lack of statistical power, and/or presence of latent nonlinear biological variables that impede the applicability of a regression approach. To address these limitations, we used machine learning to identify important features that predict adipose miR-1 change after exercise. To this end, we utilized the web-based tool for machine learning and automatic parameter tuning, CLASSify (25), to build and optimize 3 nonparametric models: K-Nearest Neighbors, Neural Network, and Logistic Regression, by grouping participants into low (< 0.5-fold; *n* = 7), medium (0.5–2-fold; *n* = 15), and high (> 2-fold; *n* = 10) change in adipose miR-1 expression after exercise (Figure 2A). Combining different machine learning models like K-Nearest Neighbors, Neural Networks, and Logistic Regression can leverage the strengths of each method, as each algorithm has a different approach to learning from data. Since different models may perform better on different parts of the data, combining models allows for better handling of different data characteristics (26). Three technical replicates were performed for each machine learning algorithm, and only the features that were consistently identified in all 3 replicates were retained (indicated by “1” in Figure 2B). Summation of the retained features among all 3 models gives the total feature score (max = 3, Figure 2B, column 1).

As expected of the positive control, the change in adipose miR-1 levels after exercise was identified by all 3 models to predict participant grouping, demonstrating the validity of the machine learning approach. In addition, other variables identified by 2 of the 3 algorithms to predict participant grouping were muscle pri-miR-1b, BMI, CD63 count at T-30, and age (Figure 2B, green boxes). The values for each of these features are plotted in Figure 2, C–F after separating into the low, medium, and high change in adipose miR-1 groups. These relationships were likely not detected with our initial classical statistical analysis due to their nonlinear nature and uneven variability, which can be appreciated most clearly for the variables of BMI (Figure 2D) and CD63 count at T-30 (Figure 2E). The positive relationship between muscle pri-miR-1b and adipose miR-1 further supports our previous findings suggesting that increased miR-1 in adipose tissue may originate from skeletal muscle in a conserved mechanism between mice and humans in response to mechanical loading (18). Furthermore, the finding that CD63^+^ EV count at T-30 predicts changes in adipose miR-1 suggests that this EV subtype might mechanistically determine the source of increased miR-1 in adipose tissue; alternatively, CD63 may be an integral contributor to EV biogenesis in the exercise response.

### RNA-Seq analyses reveal elevated EV uptake pathways in participants with high adipose miR-1 in response to exercise and identifies potential miR-1 target genes.

We previously found that, in response to mechanical overload in mice, skeletal muscle–derived miR-1 promotes lipolysis in adipose tissue by modulating β-adrenergic signaling, specifically via a transcription factor AP-2-α-CCAAT enhancer binding protein α–β adrenergic receptor 3 ((TFAP2-α-CEBP-α–ADR-β3) mechanism (18). However, in the current study, an acute bout of resistance exercise in humans did not have an effect on the expression of *ADR-*β*3*, *TFAP2-*α, or *CEBP-*α (Supplemental Figure 3). Instead, there was a significant increase in the expression of *ADR-*β*1* (*P* < 0.01) and *ADR-*β*2* (*P* = 0.02) genes in adipose tissue after exercise (Supplemental Figure 3). Notably, *ADR-*β*1* and *ADR-*β*2* predominate in human adipocytes, while *ADR-*β*3* is lowly expressed (27).

**Figure 3:**
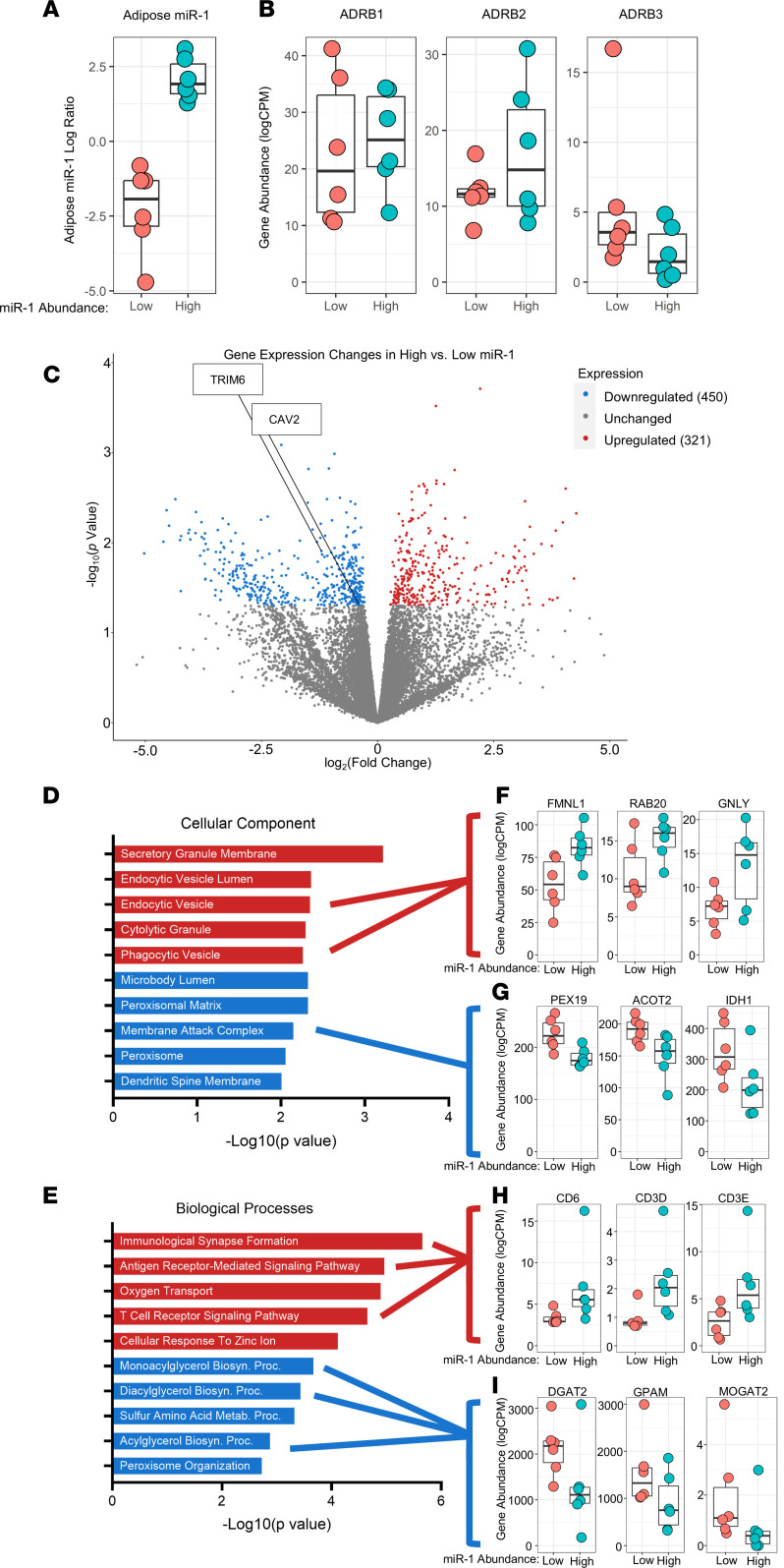
RNA-Seq analyses reveal elevated EV uptake pathways in participants with high adipose miR-1 in response to exercise and identifies potential miR-1 target genes. Expression of (**A**) miR-1, (**B**) β-adrenergic receptor 1 (*ADR-*β1*)*, *ADR-*β*2*, and *ADR-*β*3* in adipose tissue in participants with high (*n* = 6) versus low (*n* = 6) changes in miR-1. (**C**) Comparison of gene expression between high and low miR-1 responders. Gene enrichment analyses for upregulated and downregulated genes (**D** and **E**) with differences in expression levels between high and low miR-1 responders for selected genes (**F**–**I**). Data are expressed with box plots (Data are expressed with box plots displaying the 90th and 10th percentiles at the whiskers). *FMNL,* formin-like 1; *GNLY*, granulysin; *PEX19*, peroxisomal biogenesis factor 19; *ACOT2*, acyl-CoA thioesterase 2; *IDH1*, isocitrate dehydrogenase 1; *DGAT2*, diacylglycerol O-acyltransferase 2; *GPAM*, glycerol-3-phosphate acyltransferase; *MOGAT2*, monoacylglycerol O-acyltransferase 2.

To determine whether miR-1 was altering adipose gene expression, we performed bulk RNA-Seq on post-exercise adipose tissue biopsies from the 6 participants with the highest increase in miR-1 (High) compared to the 6 participants with the greatest reduction in adipose miR-1 (Low) after exercise (Figure 3A). *ADR-*β*1*, *ADR-*β*2*, and *ADR-*β*3* expression were not different (Figure 3B) between High and Low miR-1 groups, suggesting that the expression of these genes represents an exercise effect independent of miR-1 delivery in humans. RNA-Seq analysis revealed 771 differentially expressed genes (DEGs, *P* < 0.05) (Figure 3C) between High and Low adipose miR-1 groups. To gain insights in the biological and functional implications of these DEGs, we performed gene ontology (GO) analysis to identify enrichment of both cellular components (Figure 3D) and biological processes (Figure 3E). We found significant enrichment of components of secretory granule membrane, endocytic vesicle lumen, and endocytic vesicle for upregulated DEGs (Figure 3D), including formin like 1 (*FMNL1*), *RAB20*, and granulysin (*GNLY*) (Figure 3F). Downregulated DEGs were significantly enriched for components of microbody lumen, membrane attack complex, and the peroxisome (Figure 3D), including peroxisomal biogenesis factor 19 (*PEX19*), acyl-CoA thioesterase 2 (*ACOT2*), and isocitrate dehydrogenase 1 (*IDH1*) (Figure 3G). Enrichment of endocytic pathways in adipose tissue with greater increases in miR-1 suggests an important role for recipient tissues in EV-mediated communication. Further, upregulated genes were enriched for biological processes related to immunological signaling pathways (Figure 3E), including genes involved in T cell receptor signaling such as *CD6*, *CD3D*, and *CD3E* (Figure 3H). Interestingly, downregulated genes were enriched for mono- and diacylglycerol biosynthetic pathways (Figure 3G), including diacylglycerol O-acyltransferase 2 (*DGAT2*), glycerol-3-phosphate acyltransferase, mitochondrial (*GPAM*), and monoacylglycerol O-acyltransferase 2 (*MOGAT2*) (Figure 3I), suggesting negative regulation of lipid synthesis as a result of increased miR-1 in adipose tissue.

**Figure 4:**
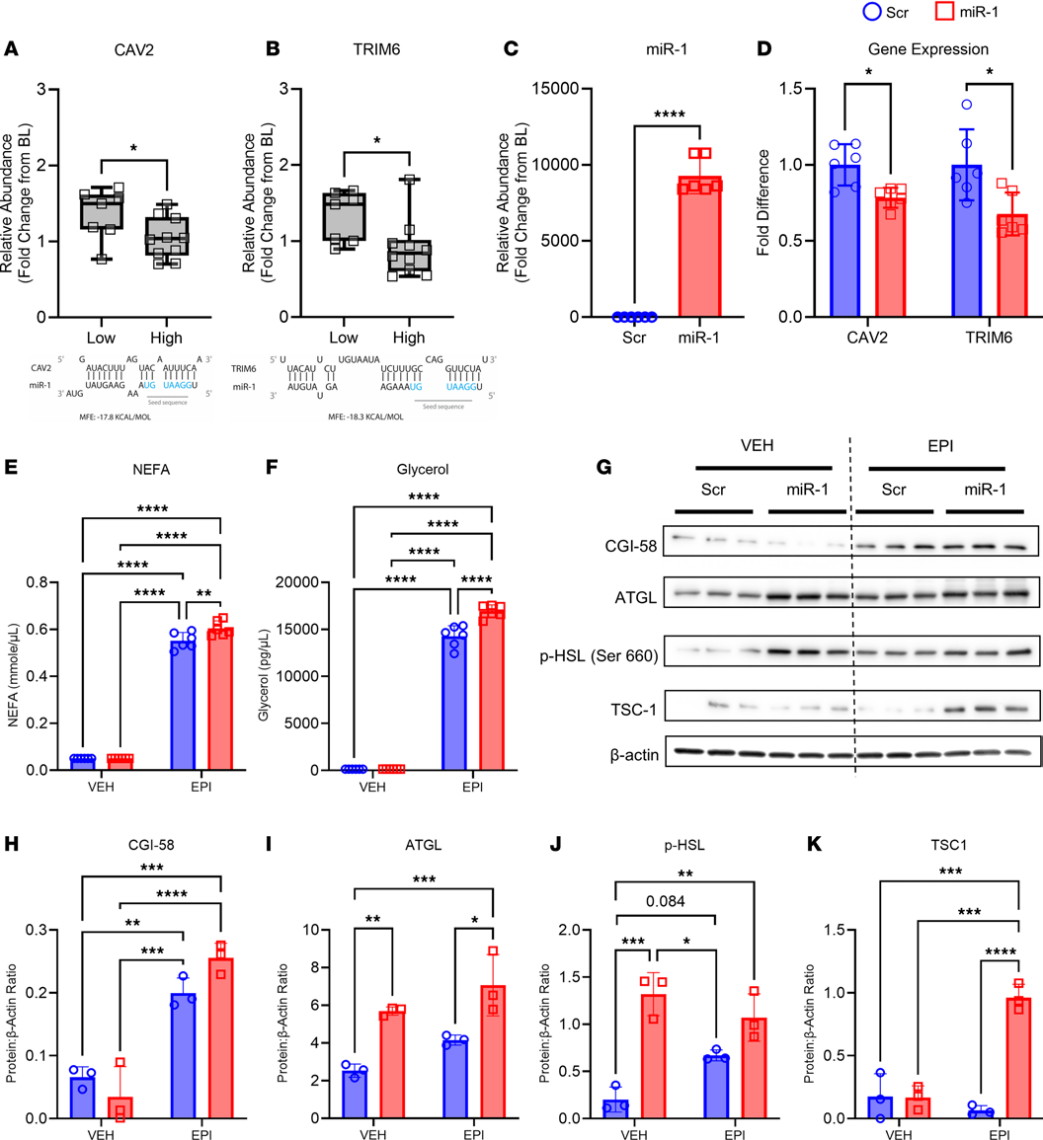
miR-1 targets CAV2 and TRIM6 mRNAs to enhance catecholamine-induced lipolysis. Differences in (**A**) caveolin 2 (*CAV2*) and (**B**) tripartite motif-containing protein 6 (*TRIM6*) expression between participants with high (*n* = 10) versus low (*n* = 7) changes in adipose miR-1 in response to exercise, as well as seed sequence schematic depicting the miR-1 binding affinity for these mRNAs. (**C**) Efficiency of miR-1 transfection as depicted by fold change in miR-1 relative to scrambled control (Scr). (**D**) Changes in *CAV2* and *TRIM6* expression with miR-1 transfection. The abundance of (**E**) Nonesterified fatty acids (NEFAs) and (**F**) glycerol in the media of scrambled control (Scr) and miR-1–transfected human adipose-derived stem cells (ADSCs) treated with vehicle (VEH) or epinephrine (EPI). (**G**) Protein abundance of (**H**) comparative gene identification-58 (CGI-58), (**I**) adipose triglyceride lipase (ATGL), (**J**) phosphorylated hormone-sensitive lipase (p-HSL), and (**K**) tuberous sclerosis protein 1 (TSC1) in Scr and miR-1–transfected ADSCs treated with VEH or EPI normalized to β-actin. Data are expressed as mean ± SD. Unpaired, 1-tailed Mann-Whitney tests were used to compare differences in CAV2 and TRIM6 expression in panel **A** and **B**. 1-tailed Welch’s unpaired *t* tests with Holm-Šidák’s corrections for multiple comparisons as needed were used to compare gene expression in panels **C** and **D**. 2-way ANOVAs with Tukey’s corrections for multiple comparisons were used to compare glycerol, NEFA, and protein levels in panels **E**, **F**, and **H**–**K**. *P < 0.05; **P < 0.01; ***P < 0.001.

Because miRs are known to exert their function through 3′-UTR recognition and RNA-induced silencing complex–mediated (RISC-mediated) repression of gene expression, we compared downregulated genes from our RNA-Seq analyses of adipose tissue of participants with High versus Low miR-1 with the miRDB database to identify 16 predicted miR-1 targets (Supplemental Table 3). Out of these DEGs, there were 2 predicted miR-1 targets that play a role in adipocyte metabolism and lipid trafficking: caveolin 2 (*CAV2*) (28–30) and a member of the tripartite motif containing protein family, *TRIM6* (31–33). In-silico prediction of miR target genes using miRanda and RNAhybrid tools to assess complementarity of the miR-1 seed sequence indicates both targets have favorable minimum free energies (MFE, ΔG°≤ –17.8 kJ) (Figure 4, A and B). Confirmatory quantitative reverse transcription polymerase chain reactions (qPCR) of adipose samples from all participants with a > 2-fold change in miR-1 (High; *n* = 10) versus those with a < 0.5-fold change in miR-1 (Low; *n* = 7) confirmed that *CAV2* (*P* = 0.02, Figure 4A) and *TRIM6* (*P* = 0.02, Figure 4B) expression were significantly lower in participants with High compared with Low miR-1. Notably, CAV2 is highly expressed in adipose tissue and is involved in lipid trafficking (30, 34) and insulin signaling (29, 35, 36), and TRIM6 is involved in the regulation of the mechanistic target of rapamycin (mTOR) C1 signaling pathway through regulating the ubiquitination of tuberous sclerosis proteins 1 and 2 (TSC1, TSC2), negative regulators of mTORC1 signaling (37). Lipid trafficking, insulin signaling, and mTORC1 signaling are heavily implicated in the regulation of lipolysis (34, 38–40). Therefore, *CAV2* and *TRIM6* represent miR-1 targets that may contribute to the regulation of lipolysis following resistance exercise.

### miR-1 targets CAV2 and TRIM6 mRNAs to enhance catecholamine-induced lipolysis.

To determine whether miR-1 regulates lipid metabolism in human adipocytes, differentiated human adipose-derived stem cells (ADSCs) were transfected with scrambled miRNA negative control (Scr) or miR-1 mimic (miR-1) to achieve robust overexpression (Figure 4C, *P* < 0.0001). The expression of the identified miR-1 targets *CAV2* *(P* = 0.02) and *TRIM6* (*P* = 0.02) were significantly reduced with miR-1 overexpression (Figure 4D), suggesting that miR-1 regulates adipocyte lipolysis through mechanisms related to lipid trafficking and mTORC1 signaling, respectively. To determine whether increasing miR-1 enhances catecholamine-induced lipolysis in human adipocytes, we treated ADSCs with vehicle (VEH) or epinephrine (EPI) after transfection with Scr or miR-1. Nonesterified fatty acid (NEFA; *P* < 0.0001) and glycerol (*P* < 0.0001) levels in the media were significantly induced by EPI treatment, with miR-1 transfection resulting in further elevations in both NEFA (interaction *P* = 0.01) and glycerol (interaction *P* < 0.0001) in EPI-treated cells (Figure 4, E and F). To provide mechanistic insight for the increase in lipolysis, we measured the expression of key proteins that control lipolysis by immunoblotting (Figure 4G). Comparative gene identification-58 (CGI-58), a positive regulator of adipose triglyceride lipase (ATGL), was induced to a higher level by epinephrine treatment in miR-1–transfected adipocytes than Scr-transfected controls (Figure 4H; interaction *P* = 0.04). ATGL catalyzes the first step in triglyceride lipolysis and was increased by miR-1 in both vehicle control- (Figure 4I; *P* < 0.01) and epinephrine-treated adipocytes (Figure 4I; *P* = 0.01). Together, the increase in ATGL and enhanced epinephrine-induction of CGI-58 by miR-1 suggest a mechanism for the increase in lipolysis by miR-1. Intriguingly, adipocytes transfected with miR-1 had higher levels of phosphorylated hormone-sensitive lipase (p-HSL; Ser660) than Scr control ADSCs in the absence of epinephrine (Figure 4J; *P* < 0.001), suggesting enhanced β-adrenergic receptor signaling by miR-1. Finally, we measured the expression of TSC1, since it is regulated by TRIM6, and would be predicted to enhance lipolysis by negatively regulating mTORC1. As shown in Figure 4K, TSC1 was highly increased by epinephrine in miR-1 transfected ADSCs (*P* < 0.0001), but not in Scr-treated ADSCs (interaction *P* < 0.001). Together, these data provide evidence that miR-1 is capable of enhancing lipolysis in human adipocytes through a complex mechanism involving increased CGI-58 and ATGL, increased HSL phosphorylation, and decreased levels of the miR-1 target *TRIM6,* leading to increased levels of the mTORC1 regulator TSC1.

## Discussion

Both chronic and acute resistance exercise have been shown to increase adipose tissue lipolysis in different human populations (41–44). However, the precise mechanism through which resistance exercise influences lipid mobilization from adipose tissue is still unclear. Increased load and heightened contraction lead skeletal muscle tissue to release factors that can influence other organs, which may explain the pleiotropic effect of resistance exercise on whole-body metabolism and body composition. The concept that skeletal muscle acts as a secretory organ is, in itself, not novel, as the field has identified numerous proteins secreted by muscle fibers during contraction, referred to as myokines (8, 45). Examples of myokines that have been shown to influence adipocyte function include IL-6 and growth and differentiation factor 15 (GDF15) (46, 47). In addition to these muscle-derived proteins with endocrine effects, emerging evidence indicates that exercise leads to secretion of EVs containing miRs that can act as lipolytic factors (17, 18). Research by our group and others indicates that exercise-like electrical pulse stimulation of myotubes leads to increased release of miR-1 into EVs (18, 48). Additionally, we previously reported that in vivo, in response to mechanical overload in mice, skeletal muscle impacts prominent cell signaling pathways in adipose tissue by delivering muscle-specific miR-1 via EVs (18). It is also notable that miR-1 is higher in the adipose tissue of insulin-sensitive individuals than insulin-resistant individuals matched for BMI (49). In the current study, we found that miR-1 primary transcript expression in skeletal muscle increases with resistance exercise in humans, concurrent with increased mature miR-1 levels in circulating EVs. Adipose tissue levels of mature miR-1 were also increased following resistance exercise, with no change in the levels of the primary transcripts for miR-1, suggesting a potential skeletal muscle origin.

The increase in miR-1 in adipose tissue following a single bout of resistance exercise may be responsible for increased lipolysis through multiple mechanisms, including lipid trafficking, insulin signaling, and mTORC1 signaling. Assessment of predicted miR-1 target genes downregulated in adipose tissue of participants with high increases in adipose miR-1 following a bout of resistance exercise identified *CAV2* and *TRIM6* as potential targets that may contribute to miR-1–mediated enhanced lipolysis. Our in vitro studies in human adipocytes confirmed that increased miR-1 levels enhance lipolysis and catecholamine-induced lipolysis. The enhancement in lipolysis with miR-1 overexpression was accompanied by reduced expression of the miR-1 targets *CAV2* and *TRIM6*. Interestingly, CAV2 is involved in lipid droplet formation and adipocyte hypertrophy, and silencing of CAV2 decreases lipid accumulation (28, 50) and increases lipolytic gene expression and glycerol release in the presence of insulin (29). Moreover, TRIM6 leads to mTORC1 activation via ubiquitination of TSC1/2, negative regulators of the mTORC1 pathway (37). mTORC1 signaling has been shown to promote lipid biosynthesis (38, 51–53) as well as restrain lipolysis (39, 53–55). Additionally, mTOR is stimulated by growth factors and insulin in adipose tissue, which act against catecholamine signaling (56). In addition to reduced TRIM6 expression in adipocytes, TSC1 protein levels were significantly higher in EPI-stimulated adipocytes in response to miR-1 overexpression. Therefore, via repression of TRIM6 and downstream inactivation of mTORC1, miR-1 may suppress fatty acid uptake and lipid storage.

Although we found that, on average, miR-1 levels increased in adipose tissue of participants after resistance exercise, there was significant variability in miR-1 changes. A potential explanation for this is the observed heterogeneity found for EV tetraspanin phenotypes. Interestingly, machine learning identified CD63^+^ EV count at T-30 as a predictor of increases in adipose miR-1 levels. Therefore, the variability in CD63^+^ EVs may be a factor explaining the heterogenous response of resistance exercise on adipose miR-1 levels. We leveraged this variability to investigate differences in gene expression between individuals with High versus Low increases in miR-1 levels. While these analyses revealed candidate miR-1 target genes, GO enrichment analysis also indicated pathways that may be related to the heterogeneity in miR-1 levels. Specifically, participants with a High adipose miR-1 response following exercise demonstrated upregulation of genes related to endocytosis. Thus, a factor that may influence adipose tissue levels of miR-1 following exercise is the intrinsic ability of recipient adipocyte cells to uptake the EVs. Differences in the capacity of cells to uptake EVs may represent a target to enhance miR delivery and lipolysis in response to resistance exercise (57). Further research is needed to elucidate the mechanisms regulating enhanced cellular EV uptake. Moreover, although we focused on miR-1, other muscle-specific miRs may also play a role in interorgan communication following resistance training. Notably, a recent study found that other miRs that target growth pathways such as mTORC1 are also increased in EVs following acute resistance exercise (58). Future research is necessary to identify other biologically active cargo contained within circulating EVs that increase following resistance exercise and to further confirm miR-1 localization in CD63^+^ EVs.

The present study has limitations. First, tissues were sampled at varying time points (i.e., 45- and 70-minutes after exercise for skeletal muscle and adipose tissue, respectively), which may have influenced our findings. The different timings in sample collection might have contributed to the divergent results observed for the miR-1 primary transcript expression and mature miR-1 levels between the 2 tissues. Second, baseline tissue sampling occurred 1 week before the exercise bout, introducing the possibility of changes in miR-1 levels during that time due to external factors unrelated to the exercise intervention. Finally, although an increase in mature miR-1 in adipose tissue with no change in the miR-1 primary transcripts suggests that the miR-1 is transferred from other tissues, the possibility that mature miR-1 is being produced in the adipose tissue (i.e., via greater processing and/or nuclear export) cannot be entirely ruled out. However, the observed increases in EV miR-1 levels and the known muscle-specificity of miR-1 (59) support a mechanism of EV-mediated transfer to adipose tissue. Still, a nonskeletal-muscle origin for the miR-1 transferred to adipose tissue also remains a possibility. Future research should further explore resistance exercise–induced communication between adipose and other tissues, including the heart.

In summary, our findings support an emerging body of literature highlighting the importance of EVs in intertissue communication. We provide compelling evidence of EV-mediated increases of muscle-specific miR-1 in adipose tissue and subsequent effects of miR-1 on cell signaling pathways leading to increased lipolysis in humans. This work adds to our understanding of the mechanisms by which resistance exercise affects adipose tissue lipolysis.

## Methods

### Sex as a biological variable.

Both male and female participants were included in this study. We report findings related to potential sex-specific effects of resistance training on EV profiles.

### Human participants.

A total of 38 relatively sedentary, exercise naive, generally healthy men and women between 18 and 39 years of age participated in the study. All participants gave informed consent and the study protocol was approved by the University of Kentucky IRB. This project was registered under ClinicalTrials ID NCT04500769. Participants were excluded if they were outside of the age range, had a history of cardiovascular, metabolic, or neuromuscular disease, or displayed evidence of infection (e.g., headache, fever, chills, shortness of breath). All participants were nonpregnant, normotensive, and nonsmokers. Participants were free of medications that could affect study outcome including, but not limited to, medications that cause excess bleeding, steroids, antiinflammatories and beta blockers. The participants were confirmed COVID-19 negative prior to muscle biopsy procedures. Six participants were unable to finish the study due to adverse effects from exercise, personal reasons, and COVID-19 infections. Therefore, 18 men and 14 women who met all inclusion criteria completed the study. Participant characteristics are provided in Supplemental Table 1.

### Experimental design.

Following screening, participants were invited to a familiarization session where they were informed about the study’s purpose and testing procedures and provided their written informed consent to participant in the study. Participants arrived to the laboratory in a fully rested, fasted state for 2 testing sessions. During the first testing session (BL), participants underwent body composition assessments, blood, skeletal muscle, and adipose collections, and performed a 1-repetition maximum (1RM) on the back squat (BS), leg press (LP), knee extension (KE), and latissimus pulldown (PD). Approximately 7 days later (7.2 ± 1.6 days), participants underwent a second testing session where an exercise bout consisting of 3 sets of 8 repetitions at 80% 1RM on BS, LP, KE, and PD, followed by a 4th set, which continued until failure. Blood was drawn immediately following the exercise bout (T-0) and at T-30, T-60, T-90. Skeletal muscle biopsies were taken again after, on average, 45 minutes and adipose tissue biopsies were taken after, on average, 70 minutes after exercise.

### Body composition.

Body composition (whole body, regional fat, and lean mass) and bone mineral density was determined using dual-energy X-ray absorptiometry (iDXA) and standardized methods for regional partitioning. All scans were analyzed by a trained and certified investigator using the GE Lunar software version 10.0. Bone mineral content (BMC; kg), bone mineral density (BMD; g/cm2), fat-free mass (FFM; kg), mineral-free lean mass (MFL; kg), fat mass (FM; kg), and BF%) were assessed.

### Blood draws.

Approximately 5–10mL was drawn at time points BL, T-0, T-30, T-60, T-90 through a single intravenous line inserted into the forearm of the participant. A licensed practitioner with extensive experience drawing blood performed all procedures. Samples were centrifuged and the serum was frozen at –80^o^C for downstream analyses.

### Skeletal muscle and white adipose tissue biopsies.

Skeletal muscle biopsies were obtained from the vastus lateralis at BL and again approximately 45 minutes after exercise under local anesthetic (1% Xylocaine HCl, 3 cc). A quarter-inch wide incision was made and a sterile 5 mm Berkstrom biopsy needle (Pelomi Industries) inserted with suction to collect 100–200 mg of skeletal muscle. Adipose tissue biopsies were obtained in similar fashion at BL and immediately after the muscle biopsy (approximately 70 minutes after exercise), resulting in the collection of 200–300 mg of tissue. A licensed practitioner with extensive experience performing skeletal muscle and adipose tissue biopsies performed all procedures. Samples were snap frozen in liquid nitrogen and stored at -80^o^C for downstream analyses.

### EV isolation and ExoView.

EVs were isolated by size exclusion chromatography using 35 nm qEV columns collecting fraction 2 (Izon Science). ExoView Human Tetraspanin Cargo kits (EV-TETRA-C; Nanoview Biosciences) were used to detect and characterize EV size, number, and surface marker expression according to manufacturer’s instructions. Isolated EVs were diluted 1:50 and loaded onto prescanned ExoView Tetraspanin chips containing spots printed with antihuman antibodies against CD63, CD81, and CD9 for EV capture, and IgG matching isotype control antibodies for nonspecific EV binding. Chips were incubated overnight in a sealed, humidified 24-well plate at room temperature for EV binding. The tetraspanin program on an ExoView CW100 plate washer (Nanoview Biosciences) was used for automated washing and fluorescently conjugated secondary antibody incubation. Antibodies against CD63, CD9, and CD81 were fluorescently labeled with excitation at 647, 488, and 555 nm, respectively. Chips were then scanned with the ExoView R100 reader using the ExoScan 3.0 acquisition software, and images were analyzed using the ExoScan 3.0 software.

### miR expression.

Total RNA was isolated from BL and post-exercise skeletal muscle and adipose tissue samples, as well as differentiated human ADSCs. Samples were homogenized using TRIzol Reagent (15596026; Thermo Fisher Scientific) in a Bullet Blender (Next Advance Inc.) and RNA was isolated using Direct-zol RNA Miniprep Plus Kits (R2070; Zymo Research). Total RNA was isolated from serum EVs at BL, T-0, T-30, T-60, and T-90 using exoRNeasy Maxi and RNeasy Plus Micro Kits (77164; 74034; QIAGEN). Reverse transcription (RT) reactions for miR-1 were performed using TaqMan MicroRNA Reverse Transcription Kit (4366596; Thermo Fisher Scientific). qPCR reactions were performed using TaqMan Fast Advanced Master Mix (4444557; Thermo Fisher Scientific) and TaqMan miRNA Assays (4427975; Thermo Fisher Scientific; miR-1 Assay ID: 002222; U6 Assay ID: 001973) in QuantStudio Real-Time PCR systems (Applied Biosystems) and miR-1 expression was determined using the 2^–ΔΔCT^ method (60). All protocols were performed according to the manufacturer’s directions.

### Gene expression.

RT reactions were performed using SuperScript VILO Master Mix (11756050; Thermo Fisher Scientific). TaqMan qPCR Assays (4427012; 4331182; 4351372; Thermo Fisher Scientific) were used to determine expression of *pri-miR-1a* (Hs03303345; Thermo Fisher Scientific) and *pri-miR-1b* (Hs03303044; Thermo Fisher Scientific) in skeletal muscle, *pri-miR-1a*, *pri-miR-1b*, *ADR*β*1-3* (Hs02330048; Hs00240532; Hs00609046; Thermo Fisher Scientific), *CEBP*α (Hs00269972; Thermo Fisher Scientific), *TFAP2*α (Hs01029413; Thermo Fisher Scientific), *CAV2* (Hs00184597; Thermo Fisher Scientific), and *TRIM6* (Hs04189681; Thermo Fisher Scientific) in adipose tissue, and *CAV2* and *TRIM6* in vitro. U6 small nuclear 1 (*U6)* expression was used for normalization for all miR targets, while the geometric mean of *U6*, glyceraldehyde 3-phosphate dehydrogenase (*GAPDH*; Hs02786624; Thermo Fisher Scientific), and 18s ribosomal RNA subunit (*18s*; Hs99999901; Thermo Fisher Scientific) was used for normalization in all gene targets. All protocols were performed according to the manufacturer’s directions.

### CLASSify.

CLASSify is a web-based tool for machine learning and automatic parameter tuning built by the Center for Applied Artificial Intelligence at the University of Kentucky, as previously described (25). We utilized this technology to build and optimize 3 nonparametric models (K-Nearest Neighbors, Neural Network, and Logistic Regression) to identify variables capable of predicting differences in the adipose miR-1 exercise response. K-Nearest Neighbors and Neural Network algorithms make no assumptions about the underlying data distribution and thus can be advantageous for handing data that does not fit traditional assumptions (61). The K-Nearest Neighbors algorithm relies on instance-based decisions, while Neural Networks leverage global pattern recognition through training and can therefore capture complex patterns. Moreover, Logistic Regression is best suited for linear relationships (61). Thus, based on the nature of the data, the predictions of these algorithms can vary.

Participants were grouped into Low (< 0.5-fold), Medium (0.5–2-fold), and High (> 2-fold) change in adipose miR-1 expression at T-90 and the models were fed descriptive (e.g., age, BMI) and experimental (e.g., skeletal muscle miR-1, EV count) variables from each participant. Three technical replicates were performed for each machine learning algorithm, and only the features that were consistently identified in all 3 replicates were retained. Feature scores (max = 3) were assigned to common variables identified by the models.

### RNA-Seq.

RNA isolated from post-exercise adipose tissue samples was used for bulk RNA-Seq. RNA-Seq was performed in adipose tissue from participants with the largest increases (High) and decreases (Low) in miR-1 abundances after exercise relative to BL (*n* = 6 per group). RNA was sent to Novogene Corporation Inc. for RNA-Seq. Downstream analyses of RNA-Seq data generated by Novogene were completed using tidyverse (version 2.0.0) (62), limma (3.54.2) (63), and edgeR (3.40.2) (64, 65) packages in R (R version 4.2.2) (66) to identify genes differentially expressed between the High and Low miR-1 responders (*P* < 0.05). Select differentially expressed genes were confirmed by comparing gene expression via qPCR (see *Gene Expression* above). Pathway analyses were performed with Enrichr (67–69); all reported pathways had *P* values < 0.01.

### In-silico miR target prediction.

A custom Python script was used to select shared miRNA: target gene interactions between miRanda and RNAhybrid tools by comparing the 3′ UTR gene region from the miR seed sequence and assessing for complementarity that suggests miR binding at the gene target. Minimal free energy was set at ΔG°= –17 kJ.

### In vitro cell culture.

ADSCs were obtained from lipoaspirate collected from healthy women undergoing liposuction and cryopreserved following 2 passages. Differentiation was induced with differentiation media containing 100 nmol/L insulin (Novolin R; Novo Nordisk Inc.), 1.0 μmol/L dexamethasone (D2915; Sigma-Aldrich), 0.25 mmol/L IBMX (I5879; Sigma-Aldrich), 0.033 mmol/L biotin, 0.017 mmol/L pantothenate (P5155; Sigma-Aldrich), and 1.0 μmol/L rosiglitazone (71740; Cayman Chemical) for 3 days, followed by 7 additional days using maintenance media without IBMX and rosiglitazone. Following 10 days of differentiation, 10 μM of mirVana miRNA Mimic miR-1 Positive Control (4464062; Ambion) or mirVana miRNA Mimic Negative Control (4464058; Ambion) was transfected into the cells for 48 hours. After the 48 hours of miRNA transfection, cells were washed with PBS twice and treated with and without 0.5 μM epinephrine (Epi) (Adrenalin; Par Pharmaceutical Inc.) in 1% BSA (A7030; Sigma-Aldrich) DMEM/Ham’s F-10 for 4 hours. Cell pellets were collected separately for downstream analyses involving RNA and protein. Media was collected from each group (miR-1^+^ Epi^+^, miR-1^+^ Epi^–^, miR-1^–^ Epi^+^, miR-1^–^ Epi^–^) and examined for nonesterified fatty acids (NEFA) (LabAssay NEFA, LABNEFA-M1; FUJIFILM Wako Healthcare Americas Corp.) and glycerol (Glycerol Assay Kit, MAK270-1KT; Sigma-Aldrich) as an assessment of lipolysis.

### Western blot analysis.

Differentiated ADSCs were lysed in a 50 mM Tris-HCL, pH 7.4, 150 mM NaCl, 1% Triton X-100, 0.1% SDS, 0.5% sodium deoxycholate, 2 mM EDTA, 50 mM sodium fluoride solution supplemented with Halt Protease & Phosphatase Inhibitor Cocktail (1861281; Thermo Fisher Scientific). Protein was subsequently isolated from miR-1^+^ Epi^–^ and miR-1^–^ Epi^–^ through centrifugation at 15,000*g* for 15 minutes at 4°C, and protein concentration was determined using Coomassie Plus Protein Assay Reagent (1856210; Thermo Fisher Scientific). For quantification of protein abundance, 20 μg of protein was loaded, resolved on a 4–15% acrylamide gel (Bio-Rad TGX Stain-Free; Bio-Rad), and transferred to a PVDF membrane. The membrane was blocked with 5% BSA in 0.1% Tween Tris-buffered saline (TBS-T) for 1 hour at room temperature, followed by a 1.5-hour incubation at room temperature with primary antibodies against ATGL (PA5-110189; Thermo Fisher Scientific), p-HSL (45804S, Ser660; Cell Signaling), CGI-58 (Ab183739; Abcam), and TSC1 (6935S; Cell Signaling) at a 1:1,000 dilution in 5% BSA. After primary antibody incubation, membranes were washed with TBS-T and incubated with appropriate goat anti-rabbit secondary antibodies (Thermo Fisher Scientific; 31460) at 1:10,000 dilution for 1 hour at room temperature in 5% BSA. Blots were developed using enhanced chemiluminescence (Clarity Western ECL Substrate, Bio-Rad) and imaged on a Chemidoc imaging system (Bio-Rad).

### Statistics.

Single-sample Wilcoxon 1-tailed *t* tests were used to compare BL and post-exercise miRNA and gene expression levels in skeletal muscle and adipose tissue. Serum miR-1 levels were compared with baseline using a 1-way ANOVA with Dunnett corrections for multiple comparisons; outliers were removed using the ROUT method (Q = 5%). Changes in EV characteristics (proportion, size, and count) from baseline to T-30 were evaluated using mixed-effects analyses with Šidák’s corrections for multiple comparisons. EV count alterations following exercise were also compared, accounting for sex and BF% using 3-way ANOVAs with Šidák’s corrections for multiple comparisons. Stepwise linear regressions were used to identify variables capable of predicting changes in adipose miR-1 with R (R version 4.2.2). Unpaired, 1-tailed Mann-Whitney tests were used to compare differences in gene expression between high and low miR-1 responders. 1-tailed Welch’s unpaired *t* tests were used to compare gene expression between scrambled and miR-1 treated cells with Holm-Šidák corrections for multiple comparisons as needed. 2-way ANOVAs with Tukey’s corrections for multiple comparisons were used to compare glycerol, NEFA, and protein levels between scrambled and miR-1 treated cells. All statistical analyses were performed using GraphPad Prism version 10.0.3 for Windows (GraphPad Software) unless otherwise noted. A *P* value less than 0.05 was considered significant.

### Study approval.

This study was conducted in accordance with the Declaration of Helsinki and approved by the Institutional Review Board at the University of Kentucky (IRB #43910). Participants received oral and written information before written consent was obtained.

### Data availability.

RNA-Seq datasets are available through Gene Expression Omnibus (GSE262576). Values for all data points in graphs are reported in the Supporting Data Values file.

## Author contributions

BIB, AI, DEL, IJV, BSF, PAK, CAP, JJM, and YW conceived and designed the experiments in this manuscript. BIB, AI, DEL, LAD, PTC, JG, TPS, BJW, IJV, BDP, TRV, CBM, HM, and YW performed the experiments. BIB, AI, DW, and YW analyzed all data. BIB, AI, BSF, PAK, CAP, JJM, and YW interpreted data. BIB, AI, and YW prepared the figures and drafted the manuscript. All authors read and approved the final manuscript. Alphabetical order of last name was used to assign authorship order for co–first authors. CP and JM were supported by the National Institutes of Health (R01DK119619). Additional funding for this study was from R01 DK124626 to PK, and the clinical research was supported by CTSA grant UL1TR001998.

## Supplementary Material

Supplemental data

Unedited blot and gel images

Supporting data values

## Version 1 09/24/2024

In-Press Preview

## Version 2 11/08/2024

Electronic publication
