# Supplementary material for: Extracellular vesicle transfer of miR-1 to adipose tissue modifies lipolytic pathways following resistance exercise
Source: JCI Insight. 2024 Nov 8;9(21):e182589. doi: 10.1172/jci.insight.182589 (PMC11601556; doi:10.1172/jci.insight.182589)
Supplement: Supplemental data [file jciinsight-9-182589-s169.pdf]

	Male		Female		Total
	Low BF%	High BF%	Low BF%	High BF%	
n	11	7	7	7	32
Race (*n*; %):<br>White<br>Black<br>Asian<br>Other	 7; 64%<br>1; 9%<br>2; 18%<br>1; 9%	 7; 100%<br>0; 0%<br>0; 0%<br>0; 0%	 6; 86%<br>0; 0%<br>1; 14%<br>0; 0%	 4; 57%<br>1; 14%<br>1; 14%<br>1; 14%	 24; 75%<br>2; 6%<br>4; 13%<br>2; 6%
Age (yr)	29.4 ± 6.9	31.3 ± 6.8	25.0 ± 3.9	31.2 ± 5.1	29.2 ± 6.2
Height (cm)	178.9 ± 7.9	181.2 ± 4.1	167.4 ± 1.8	162.7 ± 6.5	173.3 ± 9.5
Weight (kg)	78.6 ± 10.0	107.2 ± 16.4	73.3 ± 6.7	88.0 ± 20.4	85.7 ± 18.2
BMI (kg/m^2)	24.6 ± 2.8	32.6 ± 4.3	24.7 ± 4.1	33.1 ± 6.4	28.2 ± 5.9
Body Fat (%)	21.7 ± 6.3	36.8 ± 4.2	32.2 ± 4.4	44.3 ± 3.2	32.2 ± 10.0

Supplemental Table 1: *Participant characteristics.* BF% = body fat percentage.

miR-1 Relative Abundance (Fold Change from BL)	Predictors	β-Coefficient	*p*-value
Muscle	Age	-0.041	0.081
T-0 EV	Body Mass Index	0.118	0.079
T-30 EV	Bilateral Thigh Muscle Mass	0.000	0.540
T-60 EV	Bilateral Thigh Muscle Mass	0.000	0.262
T-90 EV	Estimated Visceral Adipose Mass	0.418	0.070
Adipose	Sex	0.954	0.257

Supplemental Table 2: *Low sample size precludes significant findings for predictors of changes in muscle, serum EV, and adipose miR-1.* EV = extracellular vesicle; T-0 = 0-minute timepoint; T-30 = 30-minute timepoint; T-60 = 60-minute timepoint; T-90 = 90-minute timepoint.

Gene	log(Fold Change)	*p*-value
CAV2	-0.399	0.049
CYB5B	-0.282	0.046
E2F5	-1.507	0.025
FAM169A	-2.622	0.038
GCLC	-0.420	0.023
GLCCI1	-0.733	0.013
KANK4	-1.279	0.049
MAL2	-0.917	0.039
PGD	-0.432	0.039
PPP2R5A	-0.308	0.045
RTN4IP1	-0.763	0.018
TMEM120B	-0.603	0.016
TRIM6	-1.207	0.012
ZNF112	-1.080	0.041
ZNF566	-0.397	0.045
ZNF677	-0.578	0.022

Supplemental Table 3: *Predicted miR-1 targets identified as differentially downregulated through RNA-sequencing analyses*. *CAV2* = caveolin 2; *CYB5B* = cytochrome b5 type B; *E2F5* = E2F transcription factor 5; *FAM169A* = family with sequence similarity 169 member A; *GCLC* = glutamate-cysteine ligase catalytic subunit; *GCLCI1* = glucocorticoid induced 1; *KANK4* = KN motif and ankyrin repeat domains 4; *MAL2* = T cell differentiation protein 2; *PGD* = phosphogluconate dehydrogenase; *PPP2R5A* = protein phosphatase 2 regulatory subunit B'alpha; *RTN4IP1* = reticulon 4 interacting protein 1; *TMEM120B* = transmembrane protein 120B; *TRIM6* = tripartite motif-containing protein 6; *ZNF112* = zinc finger protein 112; *ZNF566* = zinc finger protein 566; *ZNF677* = zinc finger protein 677.

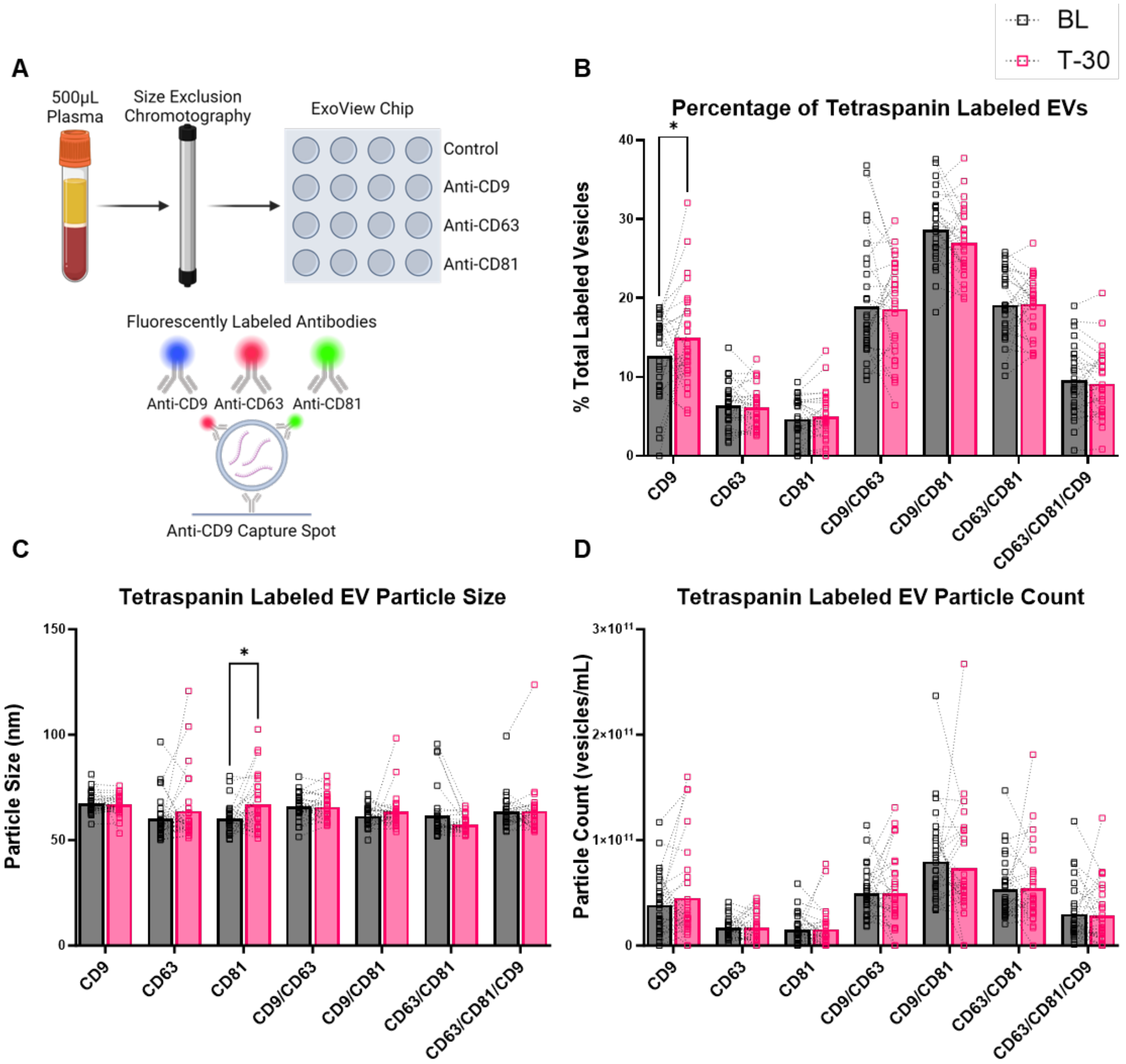

Supplemental Figure 1: *Acute resistance exercise alters extracellular vesicle (EV) size and population composition in humans*. (**A**) Schematic diagram of the workflow for the protein-capture ExoView platform. Changes in the (**B**) proportion of labeled EVs, (**C**) EV particle size, and (**D**) EV particle count in response to exercise in different populations of tetraspanin-labeled EVs (n = 29). Data were analyzed using mixed-effects analyses with Šidák corrections for multiple comparisons. * = $p < 0.05$. BL = baseline; T-30 = 30-minute timepoint.

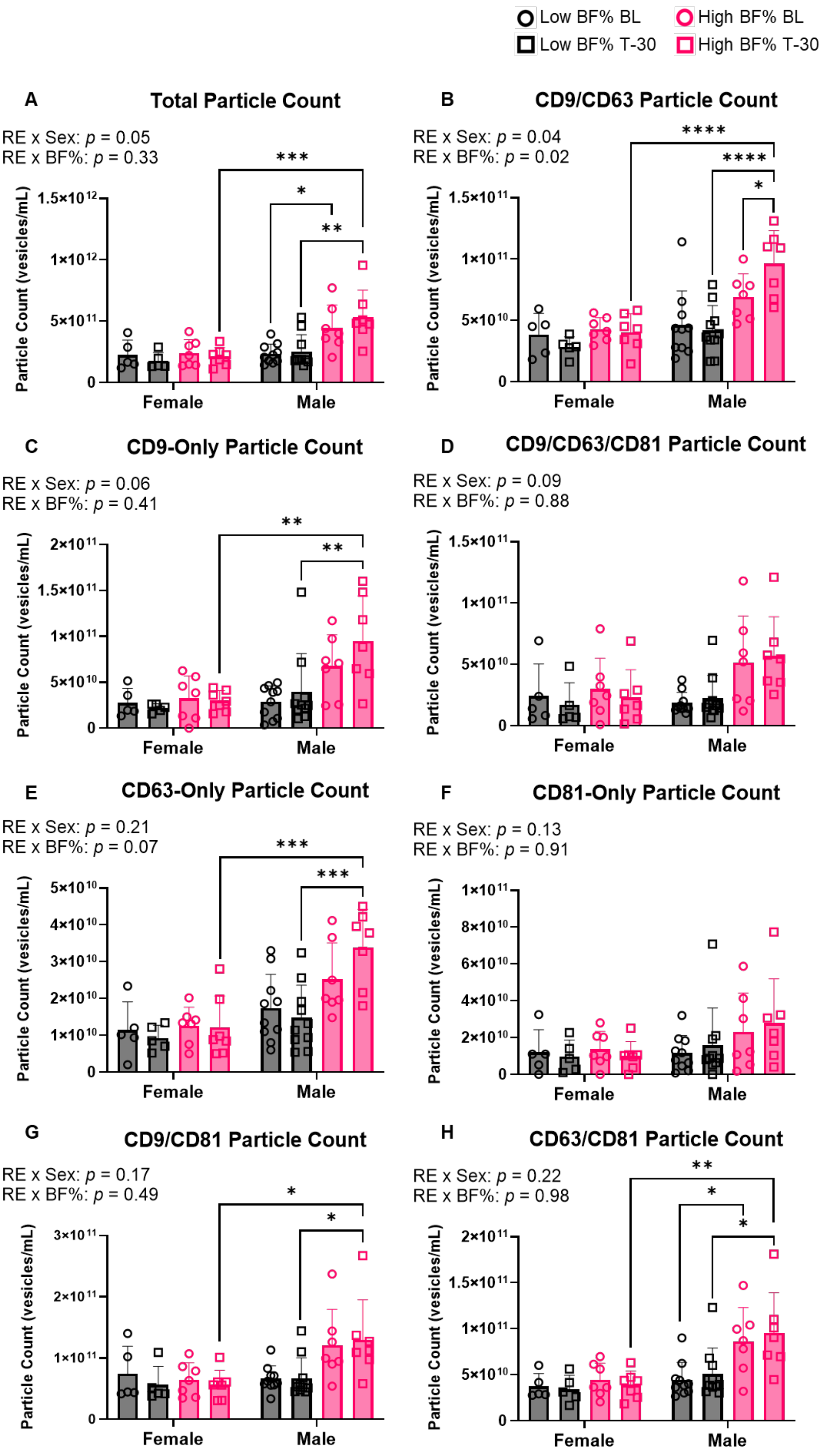
Low BF% BL
High BF% BL
Low BF% T-30
High BF% T-30
A
Total Particle Count
RE x Sex: p = 0.05
RE x BF%: p = 0.33
B
CD9/CD63 Particle Count
RE x Sex: p = 0.04
RE x BF%: p = 0.02
C
CD9-Only Particle Count
RE x Sex: p = 0.06
RE x BF%: p = 0.41
D
CD9/CD63/CD81 Particle Count
RE x Sex: p = 0.09
RE x BF%: p = 0.88
E
CD63-Only Particle Count
RE x Sex: p = 0.21
RE x BF%: p = 0.07
F
CD81-Only Particle Count
RE x Sex: p = 0.13
RE x BF%: p = 0.91
G
CD9/CD81 Particle Count
RE x Sex: p = 0.17
RE x BF%: p = 0.49
H
CD63/CD81 Particle Count
RE x Sex: p = 0.22
RE x BF%: p = 0.98
Particle Count (vesicles/mL)
Female
Male

Supplemental Figure 2: *Sex and adiposity alter extracellular vesicle response to acute resistance exercise*. Participants were groups by sex into high (male $n = 7$, female $n = 7$) and low (male $n = 10$, female $n = 5$) body fat percentage (BF%) groups. Interactions of sex and BF% with the effects of an acute bout of resistance exercise (RE) on (**A**) CD63-only, (**B**) CD9/CD63 double positive, (**C**) CD9-only, (**D**) CD9/CD63/CD81 triple positive, (**E**) total, (**F**) CD81-only, (**G**) CD9/CD81 double positive, and (**H**) CD63/CD81 double positive extracellular vesicles. Data were analyzed using three-way ANOVAs with Šidák corrections for multiple comparisons. * = $p < 0.05$; ** = $p < 0.01$; *** = $p < 0.001$; **** = $p < 0.0001$. BL = baseline; T-30 = 30-minute timepoint.

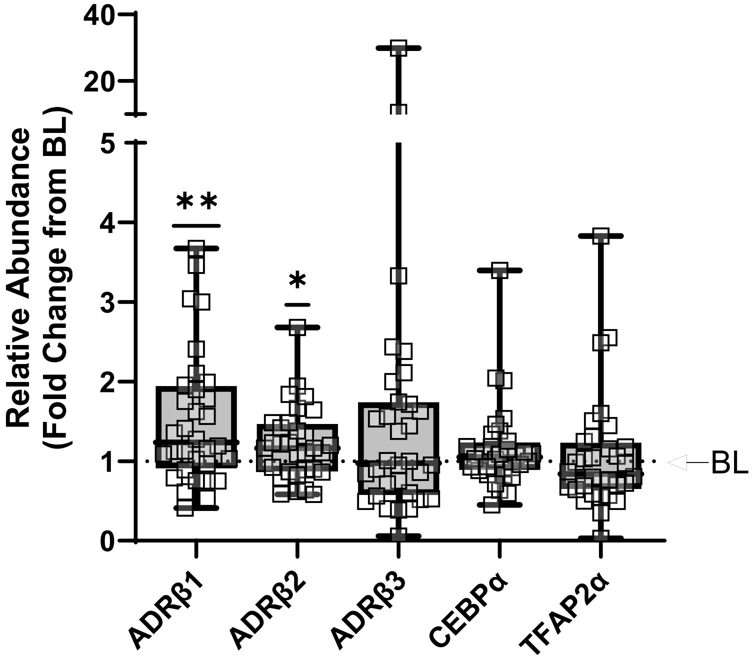

Supplemental Figure 3: *Resistance exercise increases the expression of* ADRβ1 *and* ADRβ2*, but not* ADRβ3*,* CEBPα*, or* TFAP2α. Transcript abundance of β-adrenergic receptor (*ADRβ)* 1, *ADRβ2*, *ADRβ3*, CCAAT/enhancer-binding protein alpha (*CEBPα)*, and transcription factor AP-2 alpha (*TFAP2α*) in adipose tissue relative to baseline (BL; denoted by the dotted line). Data are expressed with min-to-max box plots and were compared using one-sample Wilcoxon t-tests. * = $p < 0.05$; ** = $p < 0.01$.
