# Supplementary material for: Extracellular vesicle transfer of miR-1 to adipose tissue modifies lipolytic pathways following resistance exercise
Source: JCI Insight. 2024 Nov 8;9(21):e182589. doi: 10.1172/jci.insight.182589 (PMC11601556; doi:10.1172/jci.insight.182589)
Supplement: Unedited blot and gel images [file jciinsight-9-182589-s170.pdf]

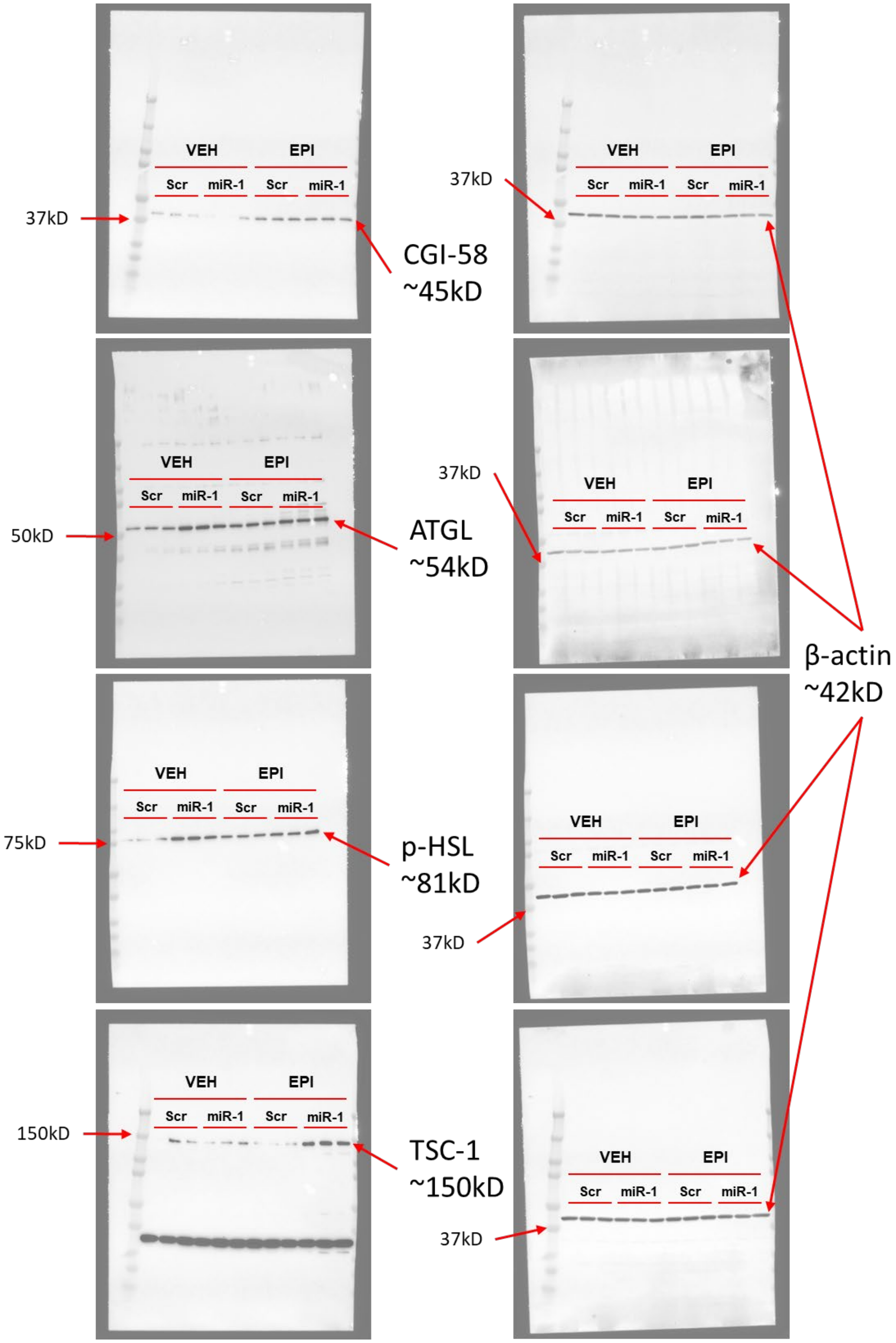

Full unedited images of western blots from Figure 4G. VEH = vehicle; EPI = epinephrine; Scr = scrambled miRNA negative control; CGI-58 = comparative gene identification-58; ATGL = adipose triglyceride lipase; p-HSL = phosphorylated hormone-sensitive lipase; TSC-1 = tuberous sclerosis protein 1.
